# Tai Chi for treating knee osteoarthritis: Designing a long-term follow up randomized controlled trial

**DOI:** 10.1186/1471-2474-9-108

**Published:** 2008-07-29

**Authors:** Chenchen Wang, Christopher H Schmid, Patricia L Hibberd, Robert Kalish, Ronenn Roubenoff, Ramel Rones, Aghogho Okparavero, Timothy McAlindon

**Affiliations:** 1 Division of Rheumatology, Tufts Medical Center, Tufts University School of Medicine, Boston, MA, USA; 2 Institute for Clinical Research and Health Policy Studies, Tufts Medical Center, Tufts University School of Medicine, Boston, MA, USA; 3 Consultant, Mind-Body Therapies Boston, MA, USA; 4 Center for Global Health Research, Tufts University School of Medicine, Boston MA, USA

## Abstract

**Background:**

Knee Osteoarthritis (KOA) is a major cause of pain and functional impairment among elders. Currently, there are neither feasible preventive intervention strategies nor effective medical remedies for the management of KOA. Tai Chi, an ancient Chinese mind-body exercise that is reported to enhance muscle function, balance and flexibility, and to reduce pain, depression and anxiety, may safely and effectively be used to treat KOA. However, current evidence is inconclusive. Our study examines the effects of a 12-week Tai Chi program compared with an attention control (wellness education and stretching) on pain, functional capacity, psychosocial variables, joint proprioception and health status in elderly people with KOA. The study will be completed by July 2009.

**Methods/Design:**

Forty eligible patients, age > 55 yr, BMI ≤ 40 kg/m^2 ^with tibiofemoral osteoarthritis (American College of Rheumatology criteria) are identified and randomly allocated to either Tai Chi (10 modified forms from classical Yang style Tai Chi) or attention control (wellness education and stretching). The 60-minute intervention sessions take place twice weekly for 12 weeks. The study is conducted at an urban tertiary medical center in Boston, Massachusetts. The primary outcome measure is the Western Ontario and McMaster Universities (WOMAC) pain subscale at 12 weeks. Secondary outcomes include weekly WOMAC pain, function and stiffness scores, patient and physician global assessments, lower-extremity function, knee proprioception, depression, self-efficacy, social support, health-related quality of life, adherence and occurrence of adverse events after 12, 24 and 48 weeks.

**Discussion:**

In this article, we present the challenges of designing a randomized controlled trial with long-term follow up. The challenges encountered in this design are: strategies for recruitment, avoidance of selection bias, the actual practice of Tai Chi, and the maximization of adherence/follow-up while conducting the clinical trial for the evaluation of the effectiveness of Tai Chi on KOA.

**Trial registration:**

ClinicalTrials.gov identifier: NCT00362453

## Background

Knee osteoarthritis (KOA) is a growing problem in the elderly, resulting in pain, functional limitations, disability and decreased quality of life leading to lost productivity and increased health care costs [1,2]. The pathophysiological basis of KOA is multifaceted and includes intra-articular inflammation and collagen degradation, impaired muscle function, reduced proprioceptive acuity and the psychological traits of chronic pain [3-6]. Currently, there are neither feasible preventive intervention strategies nor effective medical remedies for the management of KOA.

Over the past 2 decades, Tai Chi, a form of mind-body therapy, has spread worldwide for health and fitness [7]. Tai Chi combines deep diaphragmatic breathing and relaxation with many fundamental postures that flow imperceptibly and smoothly from one to the other through slow, gentle, graceful movements. Significant improvements have been reported in balance, strength, flexibility, cardiovascular and respiratory function, and reduction of pain, depression, anxiety and arthritic symptoms in a variety of patient populations including KOA [8].

Thus, Tai Chi has the potential to become a novel, logistically feasible way of providing standardized exercises with a complementary mind-body approach to the management of KOA. The physical component provides exercise that is consistent with recommendations for OA (range of motion, flexibility, muscle conditioning, and aerobic cardiovascular exercise) [9], while the mind component has the potential to increase psychological well-being, life satisfaction, and perceptions of health [10]. These effects are especially pertinent for the treatment of older adults who have OA with knee pain and poor physical function.

To date, only five randomized controlled trials (RCTs) conducted between 2000 and 2007 have compared the effect of Tai Chi with various controls in patients with OA [11-15]. The results of three RCTs suggested significant pain reduction compared to controls [11-13], but the other two found no significant changes [14,15]. Significant improvements in physical function were also reported in three RCTs compared with controls [11,13,14], but no effects were seen in the other two [12,15]. Of the two RCTs [14,15] that evaluated the effects of Tai Chi on quality of life, only one reported positive results for Tai Chi compared with controls [15]. In addition, only two RCTs reported significant differences between Tai Chi and control in improvements in flexibility or balance [11,13]. Heterogeneity of controls, different Tai Chi styles, doses and duration in addition to multiple OA sites prohibit a meaningful comparison across these trials. Furthermore, the absence of radiographic evidence of KOA as specified by the ACR criteria for OA [14], high dropout rates [13,14], small sample size [11-13,15], the lack of standardized outcome measures and short follow up [11-15] limit widespread applicability of the results from these studies.

Because the overall findings from these RCTs suggest some favorable effects of Tai Chi on pain, physical function, quality of life, balance and flexibility in patients with KOA, a well designed study may be able to overcome the limitations of the previous studies and provide a more useful treatment. We hypothesize that Tai Chi may be beneficial to patients with KOA as a result of an effect on muscle strength, flexibility, pain, stress and anxiety as well as "mind-body" interactions. We therefore designed a 12 week trial with long term 1 year follow up to obtain data on the effects of Tai Chi on pain (as a marker of disease activity), functional independence (a marker of impairment), disability, joint proprioception and health status in elderly people with KOA.

In this paper, we present the design and detailed protocol of a single-blinded, randomized controlled trial as well as a discussion of the overall challenges of conducting this trial with respect to strategies for recruitment, avoidance of selection bias, the actual practice of Tai Chi, and the maximization of adherence/follow-up. We report ways to overcome the theoretical and logistic limitations and problems of conducting such a clinical trial. The results from this trial will be reported at the completion of the study in accordance with the Consolidation of Standards for Reporting Trials guidelines [16].

## Methods/Design

### Study design

This study is a single-blinded, randomized, attention-controlled, clinical trial to evaluate the physical and psychological effects of Tai Chi for patients suffering from tibiofemoral KOA. Our goal is to compare the safety and effectiveness of Tai Chi with an attention control (wellness education and stretching program) in 40 patients with KOA. (The "single-blind" study design has been decided upon because of the inability to conceal Tai Chi assignment allocation from participants and the instructors in Tai Chi clinical trials. However, all study evaluators will be masked to treatment assignment throughout the duration of the study.)

Outcome parameters compare changes in knee pain, stiffness, and physical function using the well-validated Western Ontario and McMaster University Index (WOMAC), patient and physician global assessments, lower-extremity function, knee proprioception, depression, self-efficacy, social support, health-related quality of life, outcome expectation, adherence and occurrence of adverse events. Outcome measurements are performed at baseline, every week during the intervention period, and on completion of the 12-week program, in addition to the 24 week and 48 week follow-up. Study evaluators (study rheumatologist, study coordinator, exercise physiologist and statistician) are masked to treatment assignment.

The study setting is located in an urban tertiary care academic hospital (Tufts Medical Center) in Boston Massachusetts. The study has received Ethics approval from the Tufts Medical Center/Tufts University Human Investigation Review committee and will be conducted in the Clinical Research Center and Division of Rheumatology at Tufts Medical Center.

### Study population

This study comprises individuals with age > 55 years, Body Mass Index (BMI) ≤ 40 kg/m^2^, and with knee pain on most days of the previous month during at least one of the following activities: walking, going up or down stairs, standing upright, or in bed at night [17]. Furthermore, patients must have joint crepitus, morning stiffness lasting over 30 min. Participants are also required to have positive KOA radiological signs. Radiographic entry criteria are Kellgren and Lawrence (K/L) grade ≥ 2, defined as the presence of osteophytes in the tibiofemoral compartment and/or the patellofemoral compartment, assessed on standing anterior/posterior and lateral views (American College of Rheumatology criteria) [17]. In addition, eligible participants must have a WOMAC pain subscale score (visual analog version) of > 40 (range 0 to 500). Subjects are excluded if they have: 1. prior experience with Tai Chi or other similar types of complementary and alternative medicine such as Qi Gong, yoga or acupuncture; 2. cardiovascular or other severe disease limitations precluding full participation, as determined by the patient's primary care provider; 3. any intra-articular steroid injections in the previous 3 months or reconstructive surgery on the affected knee; 4. any intra-articular Synvisc or Hyalgan injections in the previous 6 months.

Patients can continue routine medications such as non-steroidal anti-inflammatory drugs (NSAIDS) and acetaminophen, and maintain their usual treatment visits with their primary care physician or rheumatologist throughout the study. The investigators record any changes made to treatment but do not change or recommend change in medical therapy.

### Recruitment strategies

Significant gaps in research participation do exist among ethnic minorities and thus limit the generalizability of findings. To ensure adequate enrollment of underrepresented groups, we place advertisements in the media (radio, local television, Internet, SAMPAN – a Chinese newspaper, the Boston Metro and Boston Globe newspapers). We also use the rheumatology clinic patient database at Tufts Medical Center for identifying patients with KOA. For interested respondents, the investigators provide information about the study and administer a brief, scripted interview to determine the caller's eligibility for the trial. This screening poses questions whose predictive values for KOA are known from population-based data. The lists of applicants who screen positive on the telephone interview are provided daily to the trial staff to schedule eligibility visits.

**Figure 1:**
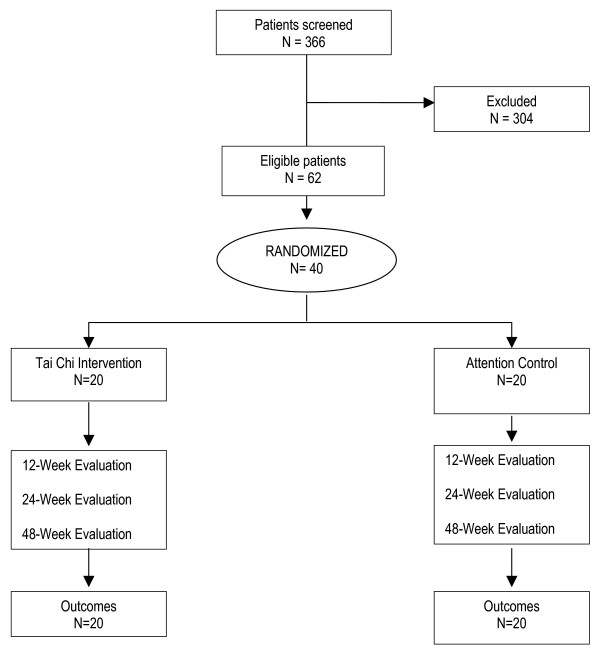
Study flow chart.

During the 5-month recruitment period, we screened 366 patients from the greater Boston area using telephone interviews from which 62 potential patients were brought into the Clinical Research Center at the Tufts Medical Center for further eligibility screening. Of these, 40 (65%) were eligible after baseline evaluation and randomized to the Tai Chi or attention control group (wellness education and stretching) (See Figure 1). The major reason for ineligibility was the absence of radiographic evidence for KOA.

### Strategies to maximize adherence

Adherence in clinical research is vital and can determine study quality and the validity of results. In order to maximize adherence, several procedures are performed: 1. Select a population of individuals who are both interested and reliable; 2. Screen patients using a well-designed questionnaire which in our previous studies consistently identified reliable individuals; 3. Discuss the proposed project in detail with each patient, especially the time commitment. A verbal and written commitment will be obtained from all participants, in which they state they will adhere to the program; 4. Schedule the visits at a time that is convenient for both patients and staff; 5. Recruit and assemble sufficient patients for baseline evaluation so that we have a large enough pool of patients to provide replacements if needed; 6. Perform the randomization after the baseline evaluation; 7. Provide friendly personal contact with participants under Institutional Review Board approval; 8. Organize entertaining Tai Chi and education intervention classes. Patients receive useful information that they could not reasonably expect to obtain through regular clinical care.

### Sample size

Our empiric sample size is guided by numbers and outcomes of an RCT conducted at Tufts University that tested an exercise intervention among older adults with KOA [18]. That study enrolled 46 patients and randomized them to either 4-month home-based progressive strength training or an attention control group of similar duration. The results were that the strength training group experienced a 36% decrease in the WOMAC index pain subscale (the primary outcome) compared to an 11% decrease in the attention control group. Thus, the exercise group had a mean change in the WOMAC pain score of -79 (SD = 91), while the control group had a mean change of -20 (SD = 77). Based on those numbers, a sample size of 20 per group and alpha = 0.05, would have power of 60% to detect a between-group difference of -59.0. While we recognize that the study is underpowered for a definitive comparison, we are primarily concerned with gathering preliminary data in order to plan and evaluate this innovative research direction.

### Randomization

Randomization assignments are made using computer-generated random numbers to randomize permuted blocks of size 2 and 4 so that each block is complete. They are provided in a sealed, opaque envelope in two groups of 10 and opened upon the patient's agreement to participate. The block size is randomly assigned to minimize correct prediction of assignments while preserving approximate balance between groups. Specially designed software developed by Tufts Medical Center is used to generate the list of random numbers and treatment assignment. Randomization envelopes are not opened unless a patient meets eligibility criteria and completes the informed consent and baseline assessment. All study envelopes are saved in the individual patient's study notebook. The statistician (CS) closely supervises the preparation process with treatment assignment packets being prepared and checked on specific days. The maximum period between screening and randomization is 3 months.

### Intervention

Forty ambulatory patients with KOA are randomly assigned to receive Tai Chi (n = 20) or attention control (wellness education and stretching) (n = 20) in twice-weekly one-hour group sessions for 12 weeks (Figure 1).

### Tai Chi

The Tai Chi program is based on the classical Yang Style [19] with some modifications as described below. Patients participate in two 60-minute Tai Chi sessions conducted weekly for 12 weeks. Each session includes: 1. warm up and review of Tai Chi principles and techniques; 2. Tai Chi exercises; 3. breathing techniques; and 4. various relaxation methods. The teachings are carried out by a Tai Chi master (RR) who has over 20 years experience conducting Tai Chi Mind-Body exercise programs. Several modifications are developed by the Tai Chi master to achieve the physical (body) and mental (mind) goals of the study for KOA, accommodate KOA symptoms and limit dropouts. For example, we eliminate the 90 degree knee-flexor joint stance used in the traditional Tai Chi exercise that would place stress on the knees and replace it by having patients rest their backs on the wall to strengthen the quadriceps muscles and also by performing sitting and standing exercises from a chair. Patients perform 2 sets: one with the legs together and one with legs apart to further strengthen various muscles around the knee. In addition, the ability to tap into the power of the mind is developed further by having patients perform visualizations while sitting on the edge of a chair. Subjects are instructed to practice Tai Chi at least 20 minutes a day at home and encouraged to maintain their usual physical activities, but not to participate in additional new strength training other than their Tai Chi exercises.

### Attention control (wellness education and stretching)

We use a wellness education and stretching program for the control group because this approach has been successfully used in other studies [18,20-22]. The program provides an active control for the attention being paid to the Tai Chi group. There is adequate personal contact with the subjects (for attention) with little anticipated effect on the main outcomes. The control groups also attend two 60-minute sessions per week for 12 weeks. Each session starts with 40 minutes of didactic sessions on 1) OA knowledge; 2) diet and nutrition; 3) therapies to treat OA; and 4) physical and mental health education (recognizing and dealing with stress and depression, etc). The final 20 minutes of the hourly session consists of stretching exercises involving the upper body, trunk and lower body, each stretch being held for 10 to 15 seconds. Subjects are also instructed to practice at least 20 minutes of stretching exercises per day at home. All subjects are encouraged to maintain their usual physical activities, but not to participate in additional strength training other than their class stretching exercises. Throughout the 12-week period, we track the number of and reasons for any missed sessions in both groups.

### Measurements

The Osteoarthritis Research Society currently recommends a core set of 4 domains (pain, physical function, patient's global assessment, and, for studies of at least 1 year, joint imaging) for outcome measurement for assessing KOA in clinical trials [17,23]. This core set is used in this study (except for joint imaging). Every participant is evaluated at baseline (prior to starting either intervention), after completing the intervention (12 weeks later), and at a 24 week and 48 week follow-up (see Table 1).

**Table 1:** Sequence of trial measurements for primary and secondary outcomes*

**VISIT**	**Baseline**	Intervention** (for 12 weeks)	**Week 12**	**Week 24 Follow up**	**Week 48 Follow up**
**Time (months)**	**-1**	**0**	**1 – 3**	**6**	**12**
**Primary outcome measure**					

WOMAC-Pain	X	X	X	X	X

**Secondary outcome measures**					

**WOMAC-Physical function**	X		X	X	X
**WOMAC-Stiffness**	X		X	X	X
**Physicians' Global KOA Severity**	X		X	X	X
**Patients' Global KOA Severity**	X		X	X	X
**SF-36 & EQ-5D & CES-D**	X		X	X	X
**Self-efficacy**	X		X	X	X
**Outcome Expectation**	X		X	X	X
**Social Support**	X		X	X	X
**Enjoyment Questionnaire**	X		X	X	X
Physical Functional Tests***	X		X	X	X
**Physical Activity Questionnaire**	X		X	X	X
**Joint Proprioception**	X		X	X	X
					
**Medications**	X	X	X	X	X
**Adverse Events**		X	X	X	X
**Body Mass Index**	X		X	X	X
**Weekly Update**		X			
**Knee X-Ray**	X				
**Adherence**		X	X	X	X
**Follow-up Questionnaire**				X	X

* KOA = Knee Osteoarthritis, WOMAC = Western Ontario and McMaster Universities Osteoarthritis Index, SF-36 = Medical Outcome Survey Short-Form 36, EQ-5D = EuroQol self-repot questionnaires, CES-D = Center for Epidemiology Studies Depression index.

** Randomization to Tai Chi or Attention Control intervention.

*** Physical Function Tests include range of motion, standing balance, 6-minute walk, and chair standing.

#### Global knee pain

The degree of self-reported global knee pain and function is recorded using the WOMAC index [24]. The WOMAC is a self-reported instrument used to assess lower extremity pain, stiffness and physical function. The primary endpoint of the study is the change in the pain subscale of the WOMAC between baseline and the 12-week assessment.

In addition, the physician provides a global score summarizing both knees using a visual analog scale version at baseline and follow up. In the baseline assessment, we use a self-reported knee-specific global pain visual analog scale (VAS) with range 0–100 mm to compare pain severity in each knee. In the event that an enrollee has identical pain scores in both knees, our statistician randomly assigns one of these to be the 'study knee' (see statistical analysis). The 'study knee' will be the knee on which primary outcome assessments are determined and used for analysis of strength outcomes.

#### Radiographs

Anterior-posterior (AP) and lateral standing knee radiographs are obtained at the initial screening examination (the baseline assessment) using the Framingham study protocol [25] and are screened by the study rheumatologist (RK) for acceptance criteria. AP knee radiographs are also obtained with the patient in the fully extended standing position and lateral images with the patients in the supine position with the knee in 30 degrees of flexion, as previously suggested [26,27]. The study rheumatologist (RK) and a musculoskeletal radiologist independently screen the radiographs using the Kellgren and Lawrence (K/L) grading system for global tibiofemoral radiographic severity, and discrepancies are resolved with a third reviewer (TM). The K/L score is determined for each knee compartment based on osteophyte formation, joint space width, and subchondral bone scleroses [28]. All scores reported are for the most severely affected knee (study knee).

#### Knee examination

Knee examination is performed at baseline and during each follow up visit. The study rheumatologist (RK), who is blinded with respect to the patient's treatment assignments, assesses the presence and severity of the knee joint abnormalities relevant to KOA. The knee examination components were selected based on recent data demonstrating the potential for reproducibility across examiners [27]. The study rheumatologist examines for swelling and tenderness of the knee joint and asks each subject to rate pain on a scale of none, mild, moderate or severe. The examination sequence is as follows: 1. Palpate study knee joint swelling (bulge sign, balloon test and patellar tap); 2. Palpate for patella tendon tenderness; 3. Palpate for patella tenderness; 4. Palpate for anserine bursa tenderness; 5. Palpate for popliteal space tenderness; 6. Rate pain in the knee/knee joint when limb is in motion; 7. Scale the general crepitus sign as none, fine or coarse; 8. Palpate for tibiofemoral tenderness laterally; 9. Palpate for tibiofemoral tenderness medially. Evidence of inflammation (e.g. joint effusion), joint deformity, and joint contractures will also be noted. Assessment of the patient's global status is also performed.

#### Range of motion (flexibility)

The passive range of motion for both knees (at full extension and flexion) is measured with a plastic goniometer (Whitehall Manufacturing, model G300,) marked in 1 degree increments. Patients are positioned on their sides with the lower extremity to be examined resting on a stabilizing board. The fixed arm of the goniometer is placed to align with the femur, pointing towards hip joint and the other arm in alignment with the fibula. With one hand holding the goniometer and supporting the leg, taking care to maintain axis of goniometer with knee axis, patients are asked to flex and extend their leg as far as they can. The maximal degrees of full flexion and extension are then read and recorded.

#### Knee joint proprioception

Knee proprioception, which is reduced in KOA [29], is measured using a Biometrics™ electrogoniometer with an ADU301 angle display unit during each assessment visit. Three test angles (30, 45 and 60 degrees) are evaluated with each subject in a sitting position taken as neutral (0 degree). The electrogoniometer is placed longitudinally in alignment with the femur and tibia with a double-sided medical tape and used to determine each of the three test angles. Patients are first shown one of the angles, which is held for a few seconds, then they are asked to close their eyes and attempt to reproduce the angle; this is repeated for all three test angles.

#### Physical performance

Physical performance assessments include the timed stand, standing balance and 6 minute walk tests. Timed stand tests measure time taken to complete ten full stands from a sitting position [30]. This is a measure of lower extremity muscle power. Patients are instructed to complete chair stand time as quickly as possible and are timed to the nearest 0.001 seconds. The same chair is utilized for testing before and after the interventions. Patients begin the chair stand seated with their arms folded across their chests, then rise to a standing position and sit back down with their back against the back rest of the chair. The test is completed when the patient stands for the 10^th ^repetition. Chair stand time is recorded using the best (lowest) score of 2 trials.

The standing balance tests include tandem, semi-tandem, side-by side, and one-legged stands. Patients are asked to maintain each position for 30 seconds. For each task, the research staff first demonstrates the task, asks the patient if they feel comfortable and ready and then supports the patient while positioning themselves [31-33]. One point is given if they exceed 30 seconds and none if they can not do or do not attempt the test.

The six minute walking test is a reliable measure of functional exercise capacity [34]. Patients are asked to walk as fast and as far as possible within the 6-minute period and are accompanied by the research staff using a wheel measure (Redi measure, Redington, Windsor, CT) that measures distance covered in inches. Patients are given verbal encouragement throughout the 6 minutes and are informed of the remaining time throughout the 6 minutes. The distance covered at the end is noted and recorded.

#### Health Related Quality of Life (HRQL)

HRQL assessments are made using the Medical Outcome Study Short Form 36 (SF-36) [35], and the EuroQol (EQ-5D) [36] instruments. The SF-36 measures 8 domains: physical functioning, role-physical, bodily pain, general health, vitality, social function, emotional health and mental health. The EQ-5D measures 5 dimensions: mobility (disability), self-care (disability), usual activities (handicap), pain/discomfort (impairment), and anxiety/depression (impairment). There is also a visual analogue thermometer rating scale to evaluate the overall patient perception of health on a 0 to 100 scale. Higher scores indicate a better health state.

#### Depression

The Center for Epidemiology Studies Depression (CES-D) index is used to assess depressive symptoms [37]. It includes a 20-item Likert-type scale with scores ranging from 0 to 60. Higher scores indicate greater dysphoria.

#### Outcome expectation

Outcome expectations are beliefs that carrying out a specific behavior such as physical activity will lead to a desired outcome. The brief outcome expectations scale (OES) [38] contains questions that ask about physical and mental benefits and will be used to assess outcome expectations. The measure is scored by summing the ratings for all the items and dividing by 9 to get the average of all 9 items. Scores can range from 1 to 5, with 1 indicating low outcome expectations for the exercise and 5 suggesting high outcome expectations.

#### Self-efficacy

Self-efficacy is important for individuals to adopt and maintain a program of regular physical activity. It is assessed using the self-efficacy instrument developed by Marcus et al [39]. The patient rates his/her confidence of being physically active in different types of situations on a 5-item scale with responses ranging from "not at all confident" to "extremely confident". The total score is computed by calculating the average of all 5 questions. A higher score indicates greater self-efficacy.

#### Social support

This is assessed using the social support for physical activity scale adapted from Cohen and colleagues [40]. Thirteen questions rated from 0 to 5 were used to assess the influences of family and friends on patients in the last 3 months as they performed regular physical activity. Higher scores reflect more perceived social support from these individuals.

#### Physical Activity Enjoyment

The Physical Activity Enjoyment Scale adapted from Kendzierski and DeCarlo [41] is used to evaluate enjoyment of physical activity. It is a self-administered 18-item, Likert scale used by patients to rate their current feelings about physical activity. The total scale score, computed after recoding, is obtained by summing the scores of all the items. High scores correspond to increased enjoyment, while low scores correspond to little enjoyment.

#### Adherence

Participants' attendance is monitored during each session for 12 weeks for both interventions via signing of attendance sheets. Patients are also asked to maintain daily Tai Chi or stretching exercise activity logs during the 12 week intervention and are encouraged with phone calls from the research staff to continue throughout the follow up period. At the 24 and 48 week visit, the research staff will ask about the average number of times per week and the number of minutes per session the subject practices at home.

#### Safety

Study patients are monitored weekly for the occurrence of adverse events defined as any undesirable experience during the duration of the study. Lack of effect of Tai Chi or stretching/education is not considered an adverse event. Patients are monitored weekly to determine whether an adverse event has occurred. All adverse events are recorded on an adverse event case report form.

### Statistical Analysis

The primary outcome is the measurement of change in knee pain between baseline and 12 weeks. Secondary outcomes are measurements of change at 24 weeks and 48 weeks. These will be analyzed both as individual time points and in longitudinal analyses. Analyses will be intention-to-treat with secondary analyses based on completers.

We will also explore a knee-based approach to the study that will use linear mixed models to adjust for correlations in outcome between knees. The most disabled knee will be used as the unit of analysis. One knee will be chosen at random if two knees are equally affected. Considering both knees would require accounting for the correlation between knees and for the presence of disability in only one knee for many participants. However, we will characterize individuals on the basis of unilateral vs. bilateral KOA and explore any possible influences of this characterization in secondary analyses.

For the patients who withdraw during the study, every effort will be made to complete a follow-up WOMAC and clinical knee examination at the time of withdrawal. If a withdrawal assessment cannot be made, we will carry forward the last assessment of knee pain, physical function or as a conservative assessment to bias towards the null hypothesis.

We will also perform a cost-effectiveness analysis to evaluate whether Tai Chi is a cost-effective therapy for patients with KOA. The costs will be estimated from health care utilization reported by subjects at the end of the trial using the Health Assessment Questionnaire that includes medication usage, hospitalization and health care professionals' visits. The differences in direct and indirect health care cost between the Tai Chi and attention control (wellness education and stretching) groups will also be compared.

## Discussion

In this article, we present the challenges of designing a randomized controlled trial with long-term follow up. The challenges encountered in this design are: strategies for recruitment, avoidance of selection bias, the actual practice of Tai Chi, and the maximization of adherence/follow-up while conducting the clinical trial for the evaluation of the effectiveness of Tai Chi on KOA.

A total of 40 eligible patients, 20 with Tai Chi and 20 with an attention control (wellness education and stretching) have already completed the study including 3 month recruitment, 12 week intervention, as well as 24 and 48 week follow-up at the Tufts Medical Center. We will perform analysis of the data and report the findings. Therefore, this project will provide important preliminary data on physical and psychological effects of Tai Chi for KOA. It will establish rigorous methods for future research for testing the mechanisms by which Tai Chi may influence pain, disability, and health related quality of life in people with KOA.

## Competing interests

The authors declare that they have no competing interests.

## Authors' contributions

CW obtained funding for the study. CW, TM, PH, RoR, CS, RK, and RaR designed the randomized controlled trial. CW, TM, PH, CS, RK and RaR conducted the research. CW and AO wrote the first draft of the manuscript. CW, TM, CS, PH, RoR, AO, RK and RaR participated in the revision of subsequent draft. All authors approved the final version of the manuscript. None of the authors declared any conflicts of financial interest.

## Pre-publication history

The pre-publication history for this paper can be accessed here:



## References

[B1] Cooper C, Klippel JH DPA (1994). Epidemiology. Rheumatology.

[B2] Rothfuss J (1997). Socioeconomic evaluation of rheumatoid arthritis and osteoarthritis: a literature review. Seminars in Arthritis & Rheumatism.

[B3] van Baar ME, Dekker J, Lemmens JA, Oostendorp RA, Bijlsma JW (1998). Pain and disability in patients with osteoarthritis of hip or knee: the relationship with articular, kinesiological, and psychological characteristics. Journal of Rheumatology.

[B4] Slemenda C, Brandt KD, Heilman DK, Mazzuca S, Braunstein EM, Katz BP, Wolinsky FD (1997). Quadriceps weakness and osteoarthritis of the knee. Annals of Internal Medicine.

[B5] Rejeski WJ, Miller ME, Foy C, Messier S, Rapp S (2001). Self-efficacy and the progression of functional limitations and self-reported disability in older adults with knee pain. J Gerontol B Psychol Sci Soc Sci.

[B6] Summers MN, Haley WE, Reveille JD, Alarcon GS (1988). Radiographic assessment and psychologic variables as predictors of pain and functional impairment in osteoarthritis of the knee or hip. Arthritis & Rheumatism.

[B7] Barnes PM, Powell-Griner E, McFann K, Nahin RL (2004). Complementary and alternative medicine use among adults: United States, 2002. Adv Data.

[B8] Wang C, Collet JP, Lau J (2004). The effect of Tai Chi on health outcomes in patients with chronic conditions: a systematic review. Archive Internal Medicine.

[B9] Jordan KM, Arden NK, Doherty M, Bannwarth B, Bijlsma JW, Dieppe P, Gunther K, Hauselmann H, Herrero-Beaumont G, Kaklamanis P (2003). EULAR Recommendations 2003: an evidence based approach to the management of knee osteoarthritis: Report of a Task Force of the Standing Committee for International Clinical Studies Including Therapeutic Trials (ESCISIT). Ann Rheum Dis.

[B10] Axford J, Heron C, Ross F, Victor CR (2008). Management of knee osteoarthritis in primary care: Pain and depression are the major obstacles. Journal of Psychosomatic Research.

[B11] Lee HY, Lee KJ (2008). Effects of tai chi exercise in elderly with knee osteoarthritis. Taehan Kanho Hakhoe Chi.

[B12] Brismee JM, Paige RL, Chyu MC, Boatright JD, Hagar JM, McCaleb JA, Quintela MM, Feng D, Xu K, Shen CL (2007). Group and home-based tai chi in elderly subjects with knee osteoarthritis: a randomized controlled trial. Clinical Rehabilitation.

[B13] Song R (2003). Effects of tai chi exercise on pain, balance, muscle strength, and perceived difficulties in physical functioning in older women with osteoarthritis: a randomized clinical trial. J Rheumatol.

[B14] Fransen M (2007). Physical activity for osteoarthritis management: A randomized controlled clinical trial evaluating hydrotherapy or Tai Chi classes. Arthritis Care & Research.

[B15] Hartman CA (2000). Effects of T'ai Chi training on function and quality of life indicators in older adults with osteoarthritis. J Am Geriatr Soc.

[B16] Hopewell S (2008). CONSORT for Reporting Randomized Controlled Trials in Journal and Conference Abstracts: Explanation and Elaboration. PLoS Medicine.

[B17] Altman R (1986). Development of criteria for the classification and reporting of osteoarthritis. Classification of osteoarthritis of the knee. Diagnostic and Therapeutic Criteria Committee of the American Rheumatism Association. Arthritis & Rheumatism.

[B18] Baker KR, Nelson ME, Felson DT, Layne JE, Sarno R, Roubenoff R (2001). The efficacy of home based progressive strength training in older adults with knee osteoarthritis: a randomized controlled trial. Journal of Rheumatology.

[B19] (1983). China Sports. Simplified "Taijiquan".

[B20] Buckelew SP, Conway R, Parker J, Deuser WE, Read J, Witty TE, Hewett JE, Minor M, Johnson JC, Van Male L, McIntosh MJ, Nigh M, Kay DR (1998). Biofeedback/relaxation training and exercise interventions for fibromyalgia: a prospective trial. Arthritis Care Research.

[B21] Buckelew SP, Huyser B, Hewett JE, Parker J, Johnson JC, Conway R, Kay DR (1996). Self-efficacy predicting outcome among fibromyalgia subjects. Arthritis Care Research.

[B22] Wang C (2003). Tai Chi improves pain and functional status in adult with rheumatoid arthritis. Arthritis & Rheumatism.

[B23] Bellamy N (1997). Recommendations for a core set of outcome measures for future phase III clinical trials in knee, hip, and hand osteoarthritis. Consensus development at OMERACT III. Journal of Rheumatology.

[B24] Bellamy N (1988). Validation study of WOMAC: a health status instrument for measuring clinically important patient relevant outcomes to antirheumatic drug therapy in patients with osteoarthritis of the hip or knee. Journal of Rheumatology.

[B25] Felson DT (1987). The prevalence of knee osteoarthritis in the elderly. The Framingham Osteoarthritis Study. Arthritis Rheum.

[B26] Chaission CE (2000). Detecting radiographic knee osteoarthritis: what combination of view is optimal?. Rheumatology.

[B27] Buckland-Wright JC (1995). Protocols for precise radio-anatomical positioning of the tibiofemoral and patellofemoral compartments of the knee. Osteoarthritis Cartilage.

[B28] Kellgren JH, Lawrence JS (1957). Radiological assessment of osteo-arthrosis. Ann Rheum Dis.

[B29] Koralewicz LM, Engh GA (2000). Comparison of proprioception in arthritic and age-matched normal knees. J Bone Joint Surg Am.

[B30] Csuka M, McCarty DJ (1985). Simple method for measurement of lower extremity muscle strength. American Journal of Medicine.

[B31] Messier SP (2000). Long-term exercise and its effect on balance in older, osteoarthritic adults: results from the Fitness, Arthritis, and Seniors Trial (FAST). J Am Geriatr Soc.

[B32] Guralnik JM, Glynn RJ, Berkman LF, Blazer DG, Scherr PA, Wallace RB (1994). A short physical performance battery assessing lower extremity function: association with self-reported disability and prediction of mortality and nursing home admission. J Gerontol.

[B33] Guralnik JM (1995). Lower-extremity function in persons over the age of 70 years as a predictor of subsequent disability. N Engl J Med.

[B34] Guyatt GH (1985). The 6-minute walk: a new measure of exercise capacity in patients with chronic heart failure. Can Med Assoc J.

[B35] Ware JE, Sherbourne CD (1992). The MOS 36-item short-form health survey (SF-36). I. Conceptual framework and item selection. Medical Care.

[B36] (1990). EuroQol--a new facility for the measurement of health-related quality of life. The EuroQol Group. Health Policy.

[B37] Kohout FJ, Berkman LF, Evans DA, Cornoni-Huntley J (1993). Two shorter forms of the CES-D (Center for Epidemiological Studies Depression) depression symptoms index. Journal of Aging Health.

[B38] Resnick B, Zimmerman SI, Orwig D, Furstenberg AL, Magaziner J (2000). Outcome expectations for exercise scale: utility and psychometrics. J Gerontol B Psychol Sci Soc Sci.

[B39] Marcus BH, Selby VC, Niaura RS, Rossi JS (1992). Self-efficacy and the stages of exercise behavior change. Res Q Exerc Sport.

[B40] Cohen S, Mermelstein R, Kamarck T, Hoberman HM, Sarason IIGSBR (1985). Measuring the functional components of social support.. Social support: Theory, research and applications.

[B41] Kendzierski D, DeCarlo KJ (1991). Physical activity enjoyment scale: Two validation studies. Journal of Sport & Exercise Psychology.

